# Alpha-1 antitrypsin augmentation therapy decreases miR-199a-5p, miR-598 and miR-320a expression in monocytes via inhibition of NFκB

**DOI:** 10.1038/s41598-017-14310-2

**Published:** 2017-10-23

**Authors:** Tidi Hassan, Chiara de Santi, Catherine Mooney, Noel G. McElvaney, Catherine M. Greene

**Affiliations:** 1 Department of Medicine, Royal College of Surgeons in Ireland, Dublin, Ireland; 2 Department of Medicine, Faculty of Medicine, UKM Medical Centre, Jalan Yaakob Latiff, Bandar Tun Abdul Razak, 56000 Kuala Lumpur Malaysia; 3 Lung Biology Group, Department of Clinical Microbiology, Royal College of Surgeons in Ireland, Dublin, Ireland; 4 School of Computer Science, University College Dublin, Dublin, Ireland

## Abstract

Alpha-1 antitrypsin (AAT) augmentation therapy involves infusion of plasma-purified AAT to AAT deficient individuals. Whether treatment affects microRNA expression has not been investigated. This study’s objectives were to evaluate the effect of AAT augmentation therapy on altered miRNA expression in monocytes and investigate the mechanism. Monocytes were isolated from non-AAT deficient (MM) and AAT deficient (ZZ) individuals, and ZZs receiving AAT. mRNA (qRT-PCR, microarray), miRNA (miRNA profiling, qRT-PCR), and protein (western blotting) analyses were performed. Twenty one miRNAs were differentially expressed 3-fold between ZZs and MMs. miRNA validation studies demonstrated that in ZZ monocytes receiving AAT levels of miR-199a-5p, miR-598 and miR-320a, which are predicted to be regulated by NFκB, were restored to levels similar to MMs. Validated targets co-regulated by these miRNAs were reciprocally increased in ZZs receiving AAT *in vivo* and *in vitro*. Expression of these miRNAs could be increased in ZZ monocytes treated *ex vivo* with an NFκB agonist and decreased by NFκB inhibition. p50 and p65 mRNA and protein were significantly lower in ZZs receiving AAT than untreated ZZs. AAT augmentation therapy inhibits NFκB and decreases miR-199a-5p, miR-598 and miR-320a in ZZ monocytes. These NFκB-inhibitory properties may contribute to the anti-inflammatory effects of AAT augmentation therapy.

## Introduction

Alpha-1 antitrypsin (AAT) deficiency is characterised by lower than normal circulating levels of AAT leading to a decreased antiprotease protective screen within the lung^1^. Although hepatocytes are the major source of circulating AAT, other cell types including monocytes can express AAT and our group has shown that accumulation of misfolded ZAAT within monocytes affects their basal and agonist-induced cytokine expression^2^. Furthermore other work of ours has demonstrated that monocytes from ZZ homozygous (AAT deficient) individuals show an altered pattern of microRNA (miRNAs) compared to MM (non-AAT deficient) monocytes^3^; miRNAs are important posttranscriptional negative regulators of gene expression.

AAT augmentation therapy involves infusion of purified human plasma AAT (60 mg per kilogram of body weight per week) and has been associated with both clinical and biochemical effects^4–13^. A number of studies have suggested that AAT also exhibits anti-inflammatory properties independent of inhibition of serine proteases in a variety of cell models. For example AAT augmentation therapy has been shown to reduce levels of chemoattractant leukotriene B4 in the airways^14^. For circulating inflammatory cells such as neutrophils, infused plasma AAT has the ability to bind them and modulate IL-8 or soluble immune complex-induced neutrophil chemotaxis^15^ and neutrophil degranulation^16^. In monocytes, AAT augmentation therapy inhibits endotoxin-induced inflammatory responses such as release of TNFα and IL-β^17^. That group also demonstrated that *in vitro* AAT inhibition of LPS-stimulated TNFα and IL-10 is mediated by a rise in cAMP and activation of cAMP-dependent protein kinase^18^. Other anti-inflammatory effects of AAT on murine and human neutrophils and in lung tissues have also been described. These include alterations in surface receptors on macrophages treated exogenously with AAT^19^and activation of protein phosphatase 2A in human alveolar macrophages, monocytes, neutrophils, airway epithelial cells, and in mouse lungs. It has been proposed that these effects counterbalance inflammatory and proteolytic responses induced by TNF signalling within the lung^20^.

Studies examining the effects of AAT deficiency and AAT augmentation therapy in circulating immune cells such as monocytes have provided insight into both the pathophysiology of the disease and non-classical anti-inflammatory properties of AAT augmentation therapy.

Whether AAT augmentation therapy affects miRNA expression has not been investigated, nor is it known if some of the anti-inflammatory effects of AAT augmentation therapy may be mediated by changes in miRNA and miRNA target protein expression. Here we identify a set of differentially expressed miRNAs in monocytes from asymptomatic MM and ZZ individuals, and show further changes in the miRNA expression profile of ZZ monocytes isolated from individuals receiving AAT augmentation therapy. We elucidate the molecular mechanisms responsible for the altered expression of a subset of these miRNAs, focussing on NFκB, and link our observations regarding altered miRNA expression to other responses potentially mediated by AAT augmentation therapy.

## Results

### Global miRNA expression is altered in ZZ monocytes from individuals receiving AAT augmentation therapy

We explored the global miRNA expression pattern in monocytes from asymptomatic MM and ZZ individuals and symptomatic ZZ individuals receiving weekly AAT augmentation therapy at Day 0 and Day 2 (48-hour post-AAT augmentation therapy) (n = 3 per group). The mean ± SE serum AAT concentrations in the MM, ZZ Day 0 and ZZ Day 2 individuals were 26.2 ± 1.7, 8.1 ± 0.8, 30.29 ± 4.7 µM AAT, respectively. For the miRNA profiling studies RNA was also isolated from THP-1 monocytic cells (a human leukaemia monocytic cell line used as a control) and all samples were examined for expression of 731 human miRNAs using the Nanostring Technologies nCounter miRNA Expression Kit. Appreciable target detection occurred for 398 miRNAs across all samples. Fifty nine miRNAs were identified that were expressed at an average difference of greater or less than 1.5 fold in (i) asymptomatic ZZ monocytes compared to MM monocytes or asymptomatic ZZ monocytes compared to ZZ monocytes receiving weekly AAT augmentation therapy at (ii) Day 0 or (iii) Day 2 (Supplementary Dataset File 01).

Importantly, 21 miRNAs were altered by more than 3-fold in asymptomatic ZZ compared to MM monocytes (Supplementary Table S1, and previously reported in^3^). MiRNAs expressed in symptomatic ZZs receiving AAT augmentation therapy at Day 2 were also compared to both symptomatic or asymptomatic ZZ monocytes not receiving AAT augmentation therapy at Day 0. Many of the miRNAs that were increased or decreased > 3-fold in symptomatic ZZ monocytes receiving AAT augmentation therapy at Day 0 (compared to asymptomatic ZZ monocytes not receiving AAT augmentation therapy) were similarly differentially expressed in ZZ monocytes receiving AAT augmentation therapy at Day 2 (*italicised* in Supplementary Table S2). Eleven miRNAs were altered >3-fold in ZZ monocytes receiving AAT augmentation therapy at Day 2 versus Day 0 (Supplementary Table S3). Of these 7 were decreased, including miR-199a-5p and miR-598.

### AAT augmentation therapy alters global gene expression

Microarray analysis was perfomed on pooled samples of (i) ZZ monocytes treated *ex vivo* with AAT versus control, and (ii) in Day 2 versus Day 0 ZZ monocytes. The microarray contained probes for over 26,000 protein coding transcripts. Of these 334 genes were commonly down regulated by AAT greater than or equal to 2-fold in both datasets. These (and their fold-change in each dataset) are listed in Supplementary Dataset 02 and 03, which includes both the probe name and the gene name to allow for isoforms. A subset of 8 genes was identified (common to both samples with an absolute fold change greater than 10 in both cases). We submitted these genes to Enrichr (gene set enrichment analysis web server)^21^ and explored the gene ontology and pathway involvement of the genes. The significantly associated gene ontology terms (adjusted p-value < 0.05) were imported to REVIGO^22^ where they were clustered based on their relatedness and any redundancy was removed. Significantly enriched pathways with adjusted p-value < 0.05 were exported from Enrichr and plotted (Genes uploaded to Enrichr: *ABLIM3, CCL11, DNAJC14, FAHD1, IL17RC, LOC100287482, RHD and TPD52L2*). This identified numerous processes with altered expression of genes (Supplementary Dataset 04). Terms with significant adjusted P values were only evident in the ‘Biological Processes’ and ‘Molecular Function’ analyses, these are plotted in Supplementary Figure 1. Pathways significantly affected by the altered genes are listed in Supplementary dataset 05 and depicted in Supplementary Figure 2A–E.

### Validation of miRNA profiling and identification of NFκB as a potential regulator of altered miRNAs

**Figure 1:**
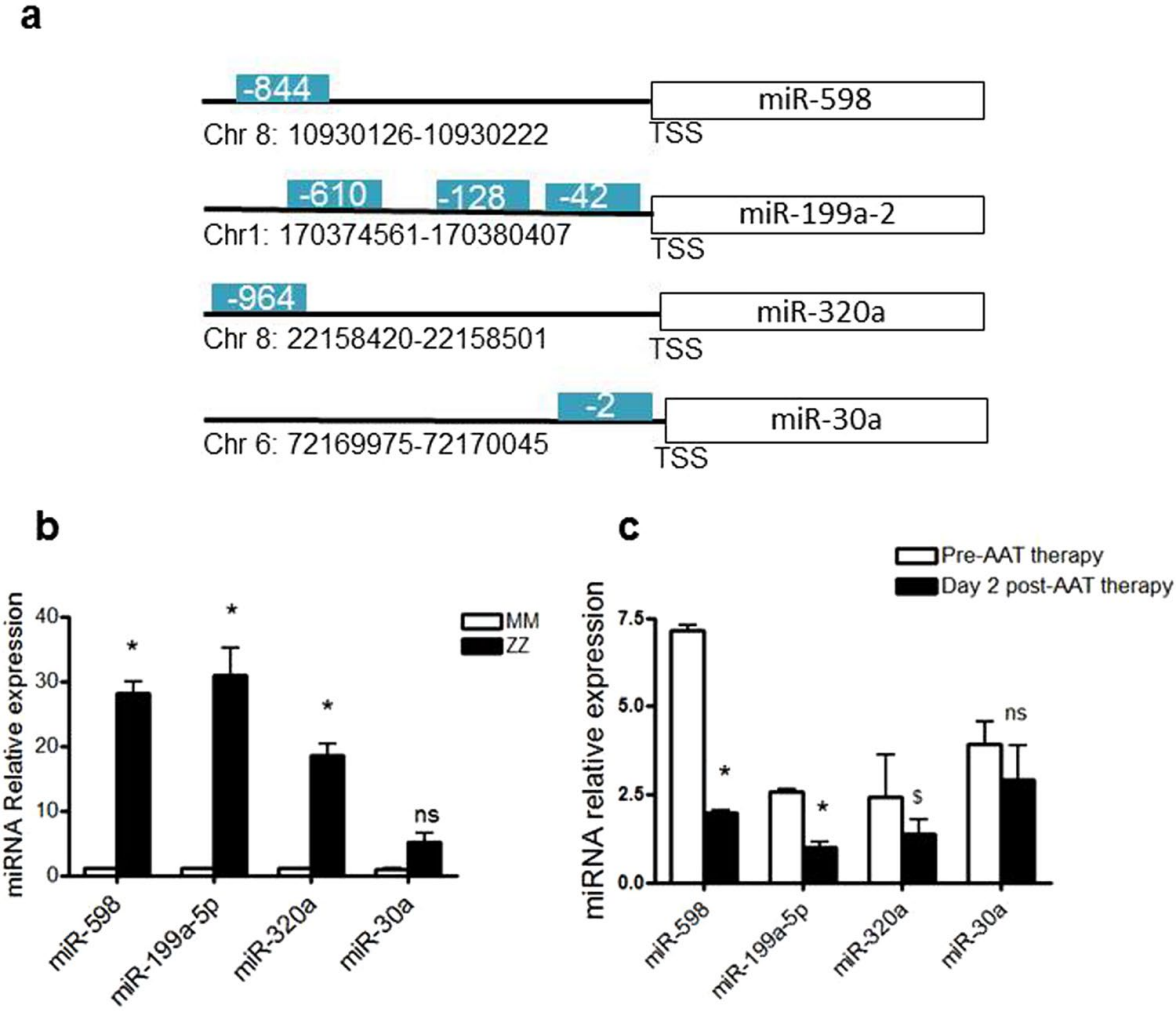
MiRNAs predicted to be regulated by NFκB are altered in ZZ individuals receiving AAT augmentation therapy. (**a**) Schematic diagram showing the location of predicted NFκB binding sites on regulatory regions of miRNA genes (TSS: transcription start site, blue box represent distance from TSS). Relative expression of miR-598, miR-199a-5p, miR-320a and miR-30a was determined using TaqMan miRNA assays and normalized to U6 snRNA using the 2^−ΔΔCt^ method in (**b**), asymptomatic MM versus ZZ monocytes (n = 4, in triplicate, *p ≤ 0.0167: t-test with Bonferroni correction for 3 test miRNAs) and (*C)*, ZZ monocytes from symptomatic individuals pre and 2 days post AAT augmentation therapy (n = 4, in triplicate, *p ≤ 0.0167; t-test with Bonferroni correction for 3 test miRNAs, ^$^p = 0.05). Data are represented as mean ± SEM.

In order to explore if the effects of AAT on miRNA expression might be mediated by modulation of transcription factors, the regulatory regions of the altered miRNAs were analysed using bioinformatics with DIANA miRGEN 2.0 (http://www.diana.pcbi.upenn.edu). This analysis highlighted that of the 21 altered miRNAs that were increased at least 3-fold in asymptomatic ZZ vs MM cells, a subset were predicted to be regulated by NFκB, namely miR-598, miR-199a and miR-320a (Fig. 1a). miR-30a-5p which contains a putative NFκB binding site in its promoter that is too close to the transcriptional start site to be functional (i.e. it is not regulated by NFκB) is also shown. This miRNA was not increased in ZZ vs. MM monocytes.

Supplementary Fig. 3A shows the heat map of these miRNAs in the samples that were profiled. The full heat map and dendogram of all altered miRNAs of interest is provided in Supplementary Fig. 3B. MiR-199a-5p was 40-fold higher in asymptomatic ZZ monocytes compared to MM monocytes. Symptomatic ZZ patients at Day 0 (i.e. 7 days after weekly AAT augmentation therapy) had 8-fold lower miR-199a-5p levels compared to asymptomatic ZZs, whilst ZZs at Day 2 had 50-fold lower levels than ZZs not receiving AAT augmentation therapy. MiR-598 and miR-320a, were also increased in ZZ versus MM monocytes (17- and 12-fold, respectively). Although AAT augmentation therapy did not alter levels of miR-320a at Day 0 and 2, or miR-598 levels at Day 0, there was a 4-fold decrease in miR-598 in ZZ monocytes receiving AAT augmentation therapy at Day 2 compared to ZZs not receiving therapy. miR-30a-5p was included as a negative control. There were no significant differences in miR-30a-5p levels in asymptomatic ZZs, MMs or ZZ monocytes receiving AAT augmentation therapy at Day 0. However, its levels increased 4-fold in ZZs at Day 2 compared to ZZ monocytes not receiving AAT.

In order to validate the miRNA profiling data, the expression patterns of these miRNAs was independently verified by qRT-PCR using Taqman miRNA assays. Similar to the profiling data, Fig. 1b shows that there was significantly increased expression of miR-199a-5p, miR-598 and miR-320a in asymptomatic ZZ compared to MM monocytes (N = 4 in each group, 22-, 28- and 16-fold increase, respectively); miR-30a was 5-fold higher.

Figure 1c shows that levels of miR-199a-5p and miR-598 were significantly decreased in symptomatic ZZ monocytes receiving AAT augmentation therapy at Day 0 and Day 2; levels of miR-320a were substantially decreased. Expression of miR-30a was not decreased following AAT augmentation therapy.

### AAT decreases miR-598, miR-199a-5p, and miR-320a in *ex vivo* ZZ monocytes

**Figure 2:**
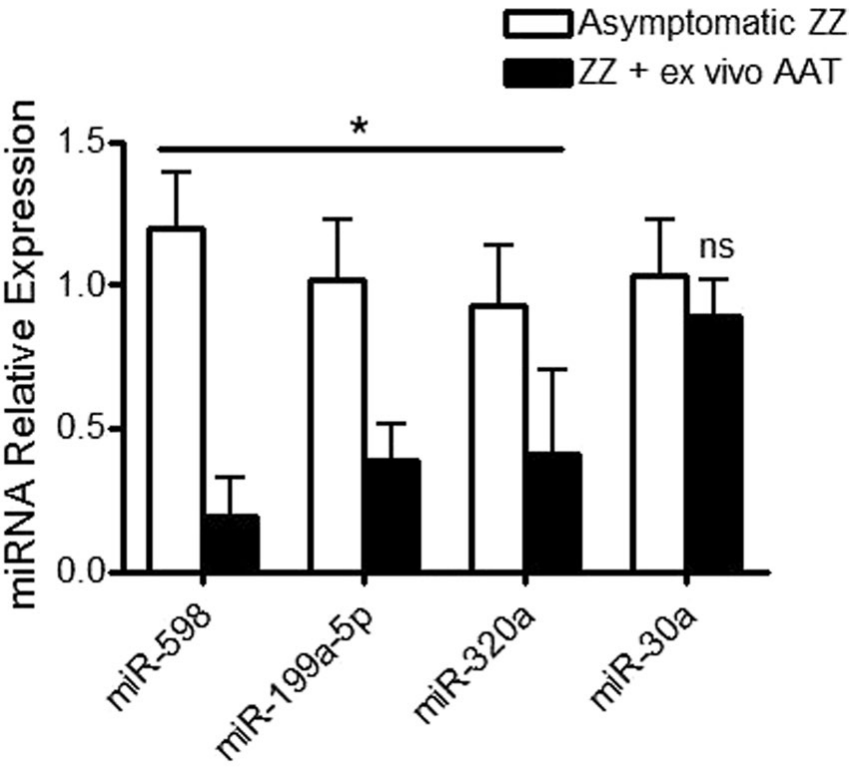
AAT decreases expression of miRNAs predicted to be regulated by NFκB in ZZ monocytes. Relative expression of miR-598, miR-199a-5p, miR-320a and miR-30a was determined using TaqMan miRNA assays and normalized to U6 snRNA using the 2^−ΔΔCt^ method in asymptomatic ZZ monocytes (1 × 10^5^ cells) and ZZ monocytes treated with 27.7 µM AAT for 4 hours. Assays were performed n = 3, in triplicate and data are represented as mean ± SEM (*p ≤ 0.0167 measured by t-test with Bonferroni correction).

The effect of exogenous AAT (27.5 µM for 4 hours) on asymptomatic ZZ monocytes was explored next. Similar to the *in vivo* observations, expression of miR-598, miR-199a-5p, and miR-320a, but not miR-30a, was significantly reduced in asymptomatic ZZ monocytes treated *ex vivo* with physiologically relevant concentrations of AAT reflecting the actual augmentation therapy dose (Fig. 2).

### Validated targets of miR-598, miR-199a-5p, and miR-320a are increased in ZZ monocytes receiving AAT augmentation therapy

**Figure 3:**
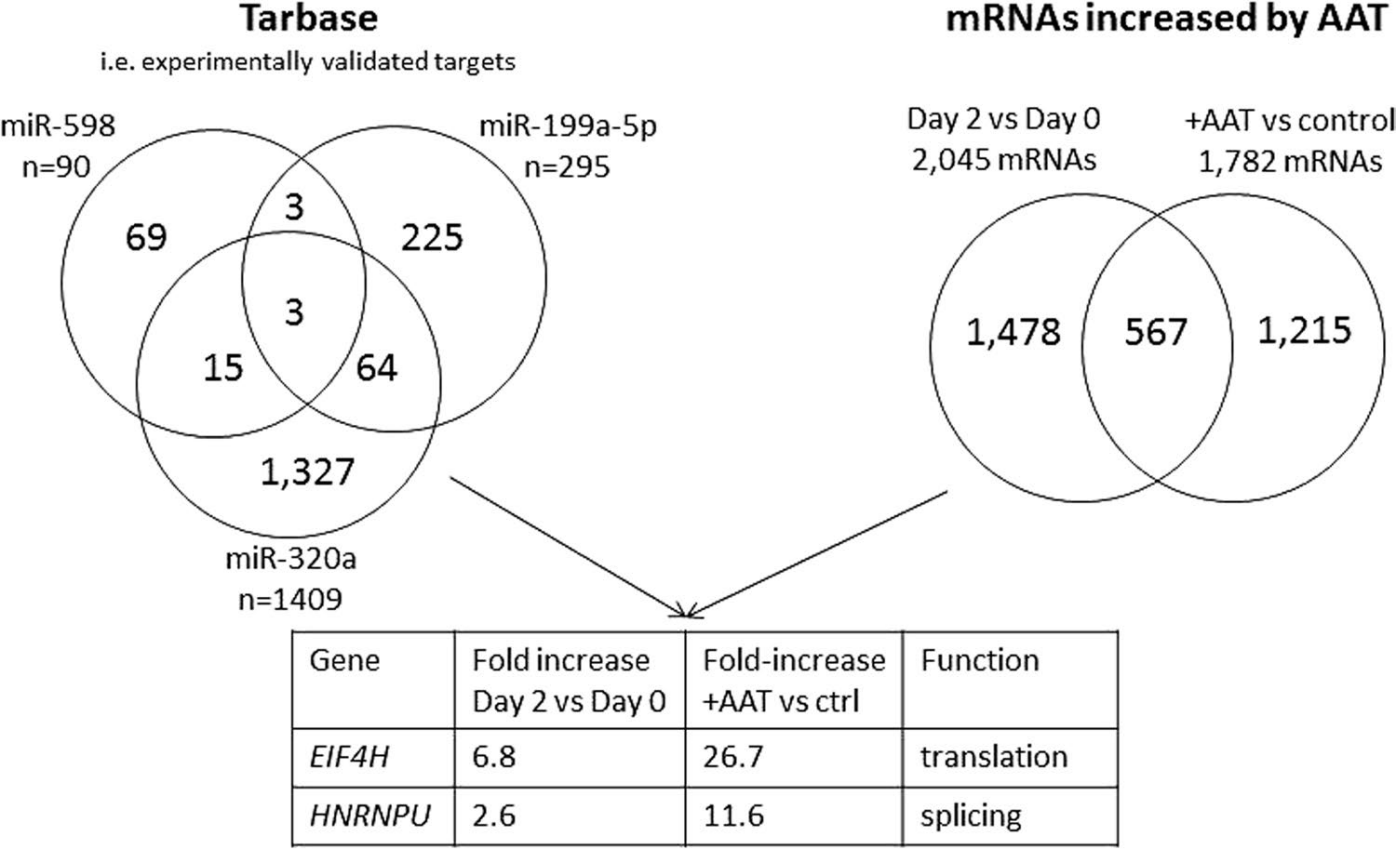
Schema to identify miRNA-mediated effects of AAT on gene expression in ZZ monocytes. *Eukaryotic translation initiation factor 4* *H* and *heterogeneous ribonuclear protein U* were identified by cross-comparing experimentally validated targets of miR-598, miR-199a and miR-320a with two microarray datasets of mRNAs upregulated in response to AAT (i.e. ZZ individuals post versus pre AAT augmentation therapy, and ZZ monocytes treated *ex vivo* with AAT vs untreated cells).

Given that AAT augmentation therapy decreased levels of miRNAs predicted to be regulated by NFκB, targets of these miRNAs are likely to be increased in response to AAT augmentation therapy. Based on this relationship we hypothesised that experimentally validated targets of miR-598, miR-199a and miR-320a, should be reciprocally increased in the same samples. Therefore we cross-compared data for each of these miRNAs alone and together with microarray data specifically focusing on mRNAs that are increased in response to AAT i.e. in ZZ individuals post- versus pre-augmentation therapy, and in ZZ monocytes treated *ex vivo* with AAT vs untreated cells (Fig. 3). We identified a selection of mRNAs that were increased in both groups known to be individually regulated by the lead miRNAs (Supplementary Tables 4–6). These deregulated mRNAs were also evaluated for possibly belonging to specific pathways and five most relevant ones (although none with significant adjusted P values) are reported in Supplementary Table 7.

Only two mRNAs that were known to be co-regulated by two of the lead miRNAs (miR-199a-3p and miR-320a) were increased in both microarray datasets; eukaryotic translation initiation factor 4 H (*EIF4H*) and heterogeneous nuclear ribonuclear protein U (*HNRNPU*) which have roles in translation and splicing, respectively. These results suggest that AAT augmentation therapy may enhance these events in monocytes. There were no targets known to be co-regulated by all three miRNAs that were increased by AAT *in vivo* or *in vitro*.

### miR-598, miR-199a-5p, and miR-320a are regulated by NFκB in ZZ monocytes

**Figure 4:**
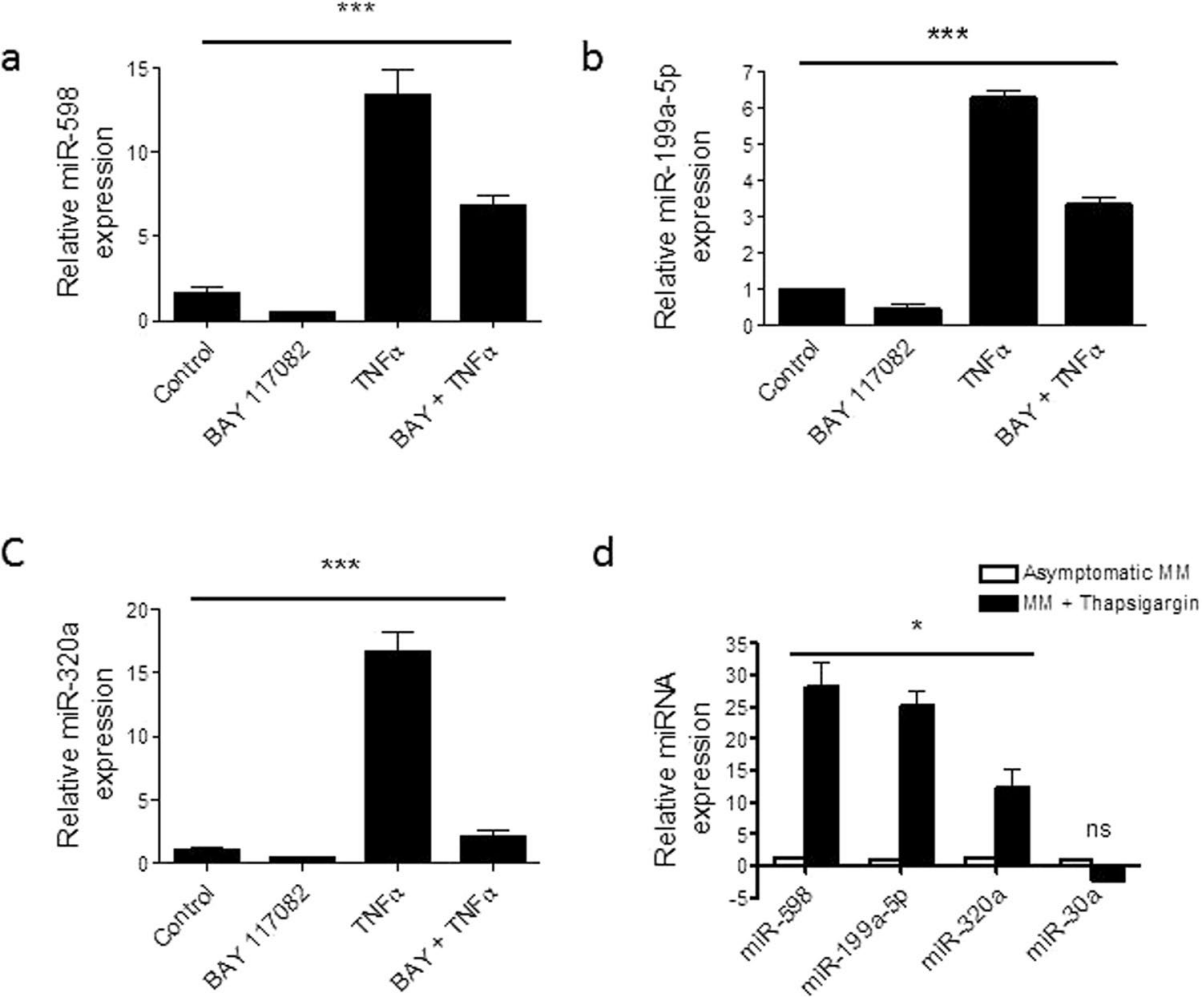
NFκB modulation regulates (**a**), miR-598, (**b**), miR-199a-5p, and (**c**), miR-320a expression. Relative expression of miRNAs normalized to U6 snRNA was determined using Taqman assays in asymptomatic ZZ monocytes (1 × 10^5^ cells, n = 3, separate cultures in triplicate) treated with or without 20uM BAY-11 7082 or 500U/ml TNF-α or both for 4 hours. (**d**), Relative expression of miR-598, miR-199a-5p, miR-320a and miR-30a was determined using the TaqMan miRNA assays in asymptomatic MM monocytes (1 × 10^5^ cells) and MM monocytes treated with 50 nM of thapsigargin for 1 hour. Assays were performed n = 3, in triplicate and data are represented as mean ± SEM (a and b ***p < 0.0001, c ***p = 0.0007 measured by ANOVA with Bonferroni’s correction for multiple group comparison. d *p ≤ 0.0167 measured by t-test with Bonferroni correction).

Having used bioinformatics to identify NFκB as a possible regulator of the altered miRNAs next we investigated the mechanism by which these altered miRNAs are decreased in response to AAT in monocytes in more detail. In order to determine if NFκB is indeed involved in the regulation of the validated miRNAs (miR-598, miR-199a-5p and miR-320a), inhibition or activation of NFκB was induced in asymptomatic ZZ monocytes by 4 hours treatment with 20 µM BAY-11 7082 or 500 U/ml TNFα, respectively (Fig. 4a–c). Monocytes treated with BAY-11 7082 showed significantly decreased expression of all three miRNAs. Basal expression of miR-30a, which was included as a negative control, was not affected by BAY-11 7082 although it was increased by TNFα (data not shown). Conversely, treatment with TNFα led to significant increases in miR-598, miR-199a-5p and miR-320a levels.

As a corollary to these investigations in ZZ monocytes, MM monocytes were treated *ex vivo* with 50 nM thapsigargin, an ER stress agonist and known activator of NFκB. Figure 4d shows that unlike the miR-30a negative control, miR-598, miR-199a-5p, and miR-328 were significantly increased in MM monocytes treated with thapsigargin compared to non-treated MM monocytes.

### NFκB p50 and p65 are down regulated in ZZ monocytes receiving AAT augmentation therapy

To further support the observation that AAT inhibits NFκB in ZZ monocytes we analysed the set of genes that were decreased in both ZZ individuals post-augmentation therapy, and in ZZ monocytes treated *ex vivo* with AAT. Of the 334 genes in this subset 294 were predicted to be regulated by NFκB (Supplementary Dataset File 06) – thus AAT appears to have a strong inhibitory effect on NFκB-regulated gene expression at least in ZZ monocytes. A Gene Set Enrichment Analysis (GSEA) on downregulated genes after augmentation therapy or AAT treatment *in vitro* using an NFκB gene signature was performed to support NFκB involvement. The full set of genes down-regulated in Day 2 vs. Day 0 (n = 3173) and down-regulated in ZZ + AAT vs. Control (n = 1760) were screened for NFκB enrichment against the hallmark gene set, which is one of the 8 major collections of gene sets in the Molecular Signatures Database (MSigDB), and is comprised of 50 gene sets. (http://software.broadinstitute.org/gsea/msigdb/genesets.jsp?collection = H). The HALLMARK_TNFA_SIGNALING_VIA_NFKB (Genes regulated by NFκB in response to TNF) gene set was the second and fifth most enriched of 50 Hallmark gene sets in “Day 2 vs. Day 0 (Normalized Enrichment Score (NES) = −1.60; FDR q < 0.17) and “ZZ + AAT vs. Control (NES = −1.34; FDR q < 0.70), respectively.

**Figure 5:**
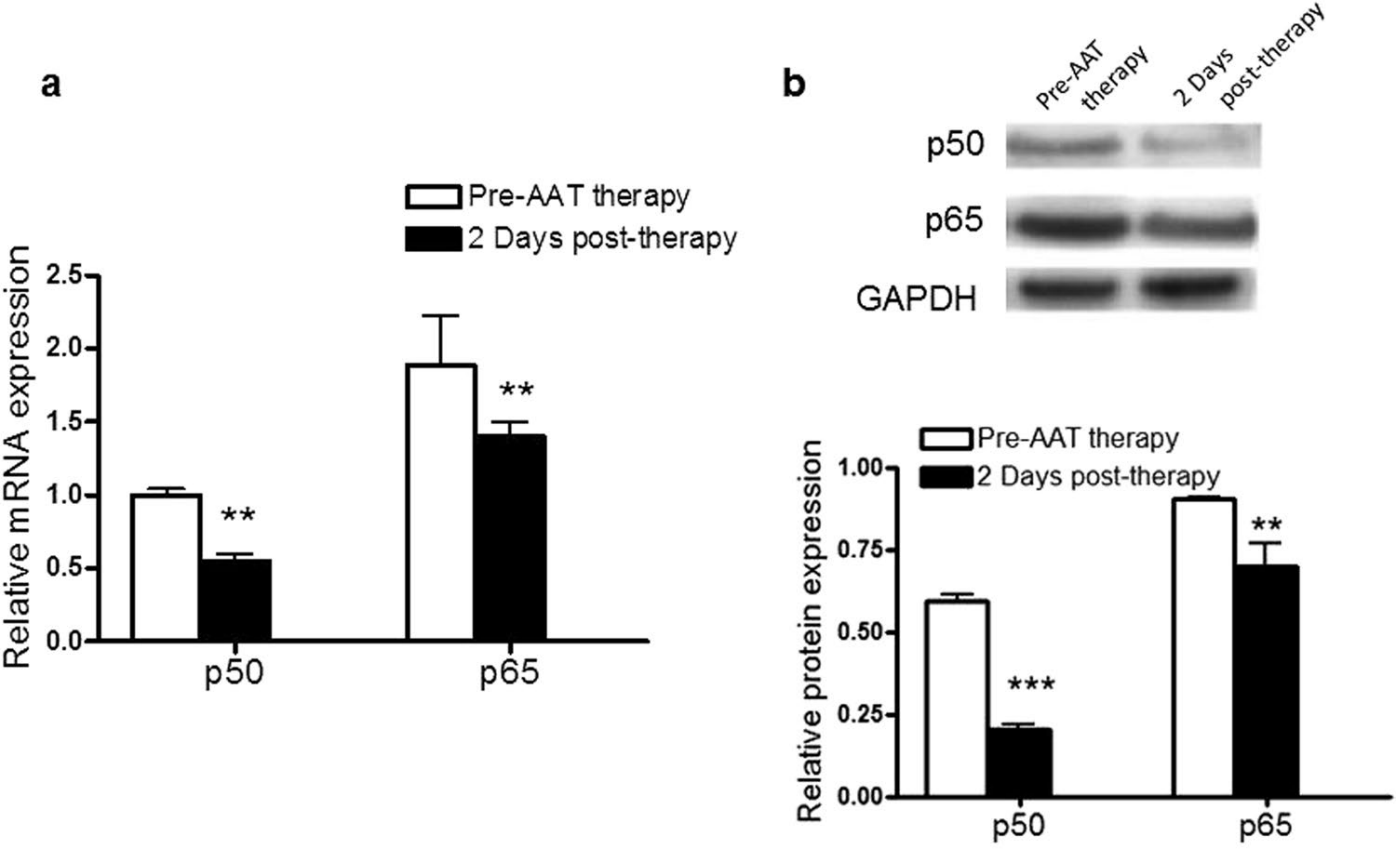
NFκB mRNA and protein expression is decreased in symptomatic ZZ monocytes receiving AAT augmentation therapy. Relative expression of p50 and p65 (**a**), mRNA and (**b**), protein in ZZ monocytes pre-AAT therapy and 2 days post AAT augmentation therapy (N = 5 in each group). mRNA and protein relative expression were analysed in triplicate by qRT-PCR and western blot respectively and normalized to GAPDH. aA representative cropped western blot in shown in b, the full-length blots are provided in the Supplementary Information File as Supplementary Fig. 4. Data are represented as mean ± SEM, **p and *** < 0.025 measured via t-test with Bonferroni correction for 2 factors).

Finally, in order to functionally link the observations regarding NFκB-regulated miRNAs and AAT augmentation therapy, the effect of intravenous AAT augmentation therapy on NFκB was determined. Expression of p50 and p65 mRNA and protein was measured using qRT-PCR and western blot analyses, respectively (Fig. 5). When compared to Day 0 ZZ monocytes, p50 and p65 mRNA levels were significantly lower in ZZ monocytes receiving AAT augmentation therapy at Day 2 (Fig. 5a). A similar observation was evident for p50 and p65 protein expression (Fig. 5b).

## Discussion

Monocyte studies in AAT deficiency have revealed the importance of circulating immune cells as effectors contributing to the inflammatory milieu in the AATD lung. Here, using an *in vivo* model of human monocytes, we report significant alterations in miRNA expression profiles following AAT augmentation therapy. Our analysis revealed that miR-199a-5p, miR-598 and miR-320a are microRNAs which are increased in ZZ vs. MM monocytes *in vivo*. All three miRNAs were also increased in MM monocytes treated with the ER stress agonist thapsigargin suggesting that these miRNAs may be ER stress-inducible miRNAs independent of ZAAT. miR-199a-5p, miR-598 and miR-320a were decreased in response to AAT augmentation therapy. Validated targets co-regulated by these miRNAs are involved in translation and splicing and their transcripts are reciprocally increased in ZZ monocytes receiving AAT. We show that NFκB regulates miR-199a-5p, miR-598 and miR-320a, and that in addition to these miRNAs, almost 300 other NFκB-regulated genes are decreased in ZZ monocytes receiving AAT. We also show that p50 and p65 mRNA and protein are lower in ZZ monocytes receiving AAT, and conclude that AAT augmentation therapy decreases expression of miR-199a-5p, miR-598 and miR-320a in ZZ monocytes via inhibiting NFκB. These events may contribute to the therapeutic effects of AAT augmentation therapy.

AAT is the most important serpin in the lung where it is responsible for the inhibition of neutrophil elastase and other serine proteinases. People with AAT deficiency, and especially those with severe AATD that are homozygous for the Z allele (Glu342Lys), have lower than normal intrapulmonary and circulating levels of AAT due to accumulation of the misfolded Z protein in the endoplasmic reticulum of hepatocytes, the principal source of AAT. The misfolding defect characteristic of ZAAT leads to the formation of polymers of ZAAT^23^. AAT augmentation therapy with purified human plasma AAT is the only established and approved therapy for AAT deficiency and is now widely used in Europe and North America. In 2015 the findings from a 2-year, placebo-controlled clinical trial (RAPID-RCT) were published demonstrating clinical efficacy of AAT augmentation therapy wherein progression of emphysema was slowed by 34%^12^. Subsequently the same patients entered a 2-year open-label extension trial (RAPID-OLE), in which all patients received AAT augmentation therapy^13^. That study confirmed the continued efficacy of AAT augmentation therapy over 4 years for the prevention of emphysema, showed that the therapy can slow lung density loss, and hence disease progression.

In addition to its serine antiprotease property AAT also has anti-inflammatory properties that can affect inflammatory cells including monocytes and neutrophils. Short-term AAT augmentation therapy not only restores airway concentrations of AAT to normal, but also reduces levels of LTB4, a major mediator of neutrophil recruitment and activation^14^. Infused AAT can bind to circulating neutrophils and modulate neutrophil migration to the airways by preventing release of CD16b from the cell membrane^15^. In monocytes, exogenous AAT can strongly inhibit LPS-induced human monocyte activation and production of IL-10^17,18^. The mechanisms responsible for these additional properties of AAT have not been fully elucidated although roles for cAMP and protein phosphatase 2 A have been clearly demonstrated^18,20^. We speculated that AAT-mediated alterations in microRNA expression may underpin some of these effects, and designed this study to address that question.

The major function of miRNAs is to negatively regulate target protein expression. Depending on the degree of complementarity been a miRNA’s seed region and its cognate miRNA recognition element within a target mRNA, mRNA degradation can be induced – this has been reported to occur for over 80% of miRNA targets^24^. Based on this fact we interrogated gene array data from two AAT-treated sample sets (compared to their non-AAT treated controls) in order to find whether experimentally validated targets of the lead miRNAs that were decreased in response to treatment with AAT demonstrated reciprocal up regulation. Whilst the mRNA of a number of validated targets known to be co-regulated by 2 or all of the lead miRNAs were increased in either sample set (data now shown), only 2 mRNAs were increased in response to AAT in both sample sets. These transcripts *EIF4H* and *HNRNPU* encode proteins that are components of a cell’s translation and splicing machinery, respectively. Ideally EIF4H and HNRNPU protein levels would have been measured here however this was not possible due to limitation of sample availablity. The HNRNPU protein is 90.5 kDa and forms part of the spliceosome C complex. It addition to forming complexes with various proteins it also contains RNA- and DNA-binding domains. The protein is essential for efficient pre-mRNA splicing in some organs and can stabilise certain cytokine mRNA transcripts^25,26^. EIF4H is a 27.4 kDa protein translation factor that was increased 3- to 30-fold in response to AAT^27^. Some studies have suggested that it can play a role in tumorigenesis in lung and other organs^28,29^. This undesirable attribute should be explored further in the context of AAT augmentation therapy.

We also explored the potential effects that AAT augmentation therapy may have via modulation of the individual lead miRNAs. miR-199a-5p and miR-598 are normally expressed at low levels in both THP-1 cells^30^ and primary human monocytes^31^ however, we have shown that they are present in greater abundance in ZZ versus MM monocytes. AAT augmentation therapy decreases their levels to within the normal range (decreases of 50- and 5 fold, respectively). miR-320a is more abundant than miR-199a-5p and miR-598, and has been more intensively studied and therefore more of its validated targets are known. We observed a number of novel pathways can be affected by these miRNAs with the most interesting regarding what is known about AAT deficiency being ER protein processing due to altered expression of *MAN1A2*, *HSP90AA1* and *EDEM1*, validated targets of miR-598, miR-199a-5p and miR-320a, respectively.

We observed some interesting gene expression changes overall in response to AAT *in vivo* and *in vitro* which may or may not be related to alterations in miRNA expression. Significantly altered GO terms included such processes as leucocyte chemotaxis, responses to cytokines, ion transport and actin filament polymerisation. Pathways affected by these changes include IL-4 and IL-17 cytokine signalling pathways and various G-protein coupled receptor pathways including CXCR3.

Here, we also demonstrated for the first time that AAT augmentation therapy reduces the expression of both the mRNA and protein of the canonical heterodimer of NFκB in ZZ monocytes. NFκB activation in AAT deficiency and in the context of ER accumulation of misfolded ZAAT have been reported in a variety of cell types including monocytes, 16HBE14o- human bronchial epithelial cell lines, Chinese hamster ovary (CHO) cells, liver cell lines and liver cells from transgenic mice with liver-specific inducible expression of ZAAT^2,32,33^. Our data indicates that augmentation therapy can reverse this effect in ZZ monocytes. A strong inhibitory effect of AAT augmentation therapy on NFκB was evident in ZZ monocytes *in vivo* and we further confirmed this observation *in vitro*. However the mechanism by which exogenous, infused AAT may directly influence NFκB expression remains unanswered and is beyond the scope of this study. Interestingly the NFκB subunits p50 and p65 are validated targets of miR-199a-5p^3^. The present study demonstrates that NFκB is a transcription factor for miR-199a-5p suggesting that an autoregulatory loop exists between NFκB and miR-199a-5p.

The gene array studies and GSEA indicated that up to 300 genes predicted to be regulated by NFκB showed decreased expression in ZZ monocytes from individuals receiving AAT augmentation therapy on Day 2 after infusion and also in ZZ monocytes treated *ex vivo* with therapeutically relevant levels of AAT compared to matched controls. Future independent validation and functional analysis of the roles of these altered genes can shed light on unexplored biological effects of AAT augmentation therapy. Furthermore, this information coupled with extended studies to determine the consequences of AAT augmentation therapy on altered miRNA expression (via mechanisms other than inhibition of NFκB) will generate further insight into the properties and effects of AAT augmentation therapy. In conclusion the data presented here provides new mechanistic evidence regarding the potential biological effects of AAT augmentation therapy mediated by NFκB inhibition and/or altered miRNA expression that has implications for AAT deficiency and other diseases where AAT augmentation therapy may have relevance.

## Methods

### Study populations

Asymptomatic non-AAT deficient MM (n = 5, 3 males, 2 females) and AAT-deficient ZZ (n = 6, 3 males, 3 females) individuals, and ZZ individuals receiving AAT augmentation therapy (Zemaira from CSL Behring, n = 5, 3 males, 2 females) were recruited in this study. Asymptomatic MM individuals were control individuals with no evidence of any disease, respiratory symptoms and not on any medication. All controls had an MM phenotype, with serum AAT concentration within the normal range (25 to 50 µM) (mean age 29.82 ± 4.26). Asymptomatic ZZ individuals (mean age 32.18 ± 2.98) and symptomatic ZZ individuals receiving AAT augmentation therapy (mean age 49.91 ± 3.87) were recruited from the Irish AAT deficiency registry. The term ‘symptomatic’ indicates evidence of symptoms and signs of COPD with pulmonary function tests showing an obstructive pattern i.e. FEV1/FVC < 70%. Asymptomatic ZZ individuals were clinically stable, with no evidence of COPD symptoms or exacerbations in the previous 6 months. Mean forced expiratory volume in 1 second (FEV1) were were 74.13% ± 12.45 and 96.12% ± 6.15% predicted for symptomatic and non-symptomatic ZZ individuals respectively. ZZ individuals receiving AAT augmentation therapy were receiving plasma-purified AAT from CSL Behring (Zemaira), administered intravenously at a dosage of 60 mg/kg body weight weekly. Thus, in this study the term Day 0 refers to ‘pre-AAT augmentation therapy’ but also implies ‘Day 7’ whilst Day 2 refers to ‘48 hours post-AAT augmentation therapy’. Patients were free from exacerbations at least 4 weeks prior to obtaining blood samples. All ZZ individuals had a ZZ phenotype determined by isoelectric focussing, with serum concentrations of AAT less than 11 µM and confirmed with allele-specific PCR. All methods were carried out in accordance with relevant guidelines and regulations of the research ethics committee of Beaumont Hospital Dublin who approved this study. Full informed consent was obtained from all subjects.

### Isolation, culture and treatment of peripheral blood monocytes

Mononuclear cells were isolated using heparinized venous peripheral blood and treated with Lymphoprep (Axis Shield) for density gradient centrifugation. The mononuclear cell band was aspirated, washed with HBSS (Invitrogen) and monocytes were purified using the EasySep Human CD14 Selection Cocktail (StemCell Technologies). Cells were maintained in RPMI 1640, 10% (v/v) fetal calf serum (Life Technologies) and 1% penicillin/streptomycin (Invitrogen) at 37 °C in a 5% CO_2_ atmosphere. To induce endoplasmic reticulum (ER) stress, MM monocytes were treated with DMSO (vehicle control) or thapsigargin at 50 nM for 1 hour. For NFκB agonist and antagonist experiments, MM monocytes were treated with 500 U/ml TNFα or 20 µM BAY-11 7082 for 4 hours respectively. Treatment with exogenous AAT (Athens Research & Technology) at 27.5 µM for 4 hours was performed to validate the effects of AAT replacement *ex vivo*.

### miRNA and mRNA expression profiling

miRNA (40 ng) was prepared using the nCounter miRNA Sample Preparation Kit (Nanostring Technologies) according to manufacturer’s instructions (n = 3 in each group) and profiled commercially with the nCounter miRNA Expression Assay. Raw data was normalized based on the relative number of positive and negative control counts and adjusted for probe and background corrections for each miRNA as instructed in the nCounter Data Analysis Guidelines. Significance was calcuated using non-parametric permutation tests with a p-value of permutation < 0.003 considered statistically significant.

mRNA expression profiling was performed by Arraystar, Inc. (Rockville, MD, USA) on RNA from THP-1 cells or monocytes isolated from MMs, asymptomatic ZZs, symptomatic ZZs on Day 0 or Day 2, and asymptomatic ZZ monocytes treasted and not treated with 27 µM AAT for 4 hours (pooled n = 3 each). For microarray analysis an Agilent Array platform was employed and analysed as described in McKiernan *et al*.^34^. Approximately 26,109 coding transcripts collected from RefSeq (release 55), UCSC Human (GRCh37/hg19) and GENCODE 13 were detected. Differentially expressed (DE) mRNAs between two groups were identified through volcano plot filtering, and hierarchical clustering was performed to show distinguishable mRNA expression patterns among samples.

### Quantitative assessment of mRNA and miRNA levels

TRI reagent was used to isolate RNA according to the manufacturer’s instructions and 500 ng was reverse transcribed into cDNA using the Quantitect Reverse Transcription Kit (Qiagen). Oligonucleotide primers were synthesized (MWG Operon) and quantitative PCR reactions were performed in 20 µl containing 2 µl of template cDNA, SYBR Green MasterMix (Roche) and 10 pmol of each primer. For miRNA expression 100 ng of RNA was used in Taqman miRNA assays (Applied Biosystems) according to manufacturer’s instructions. A Roche LC 480 Lightcycler was used for amplification of both mRNA and miRNA in triplicate. Relative expression of mRNA and miRNA relative to GAPDH and U6 snRNA respectively was determined using the 2^−ΔΔCt^ method.

### Western blot analyses

Equal volumes of cell lysates were separated by NuPage ® Novex 4–12% Bis-Tris Gels (Life Technologies) in MOPS SDS running buffer (Life Technologies) and transferred onto nitrocellulose membranes (Sigma-Aldrich). Membranes were probed with primary antibodies (rabbit anti-p50, Santa Cruz, 1:1000 dilution or mouse anti-p65, Santa Cruz, 1:1000 dilution) and signal detection was determined using the Immobilon® Western HRP Substrate (Milipore) on the Syngene G:Box chemi XL gel documentation system. Densitometry was analysed using GeneTools software on the same system.

### Bioinformatic analyses

Diana-miRGen 2.0 was used to analyse miRNA genomic information for predicted transcription factors binding sites in miRNA genes. NFκB binding sites in genes commonly downregulated *in vivo* on Day 2 vs Day 0, and in ZZ monocytes treated *ex vivo* with AAT vs. untreated ZZ monocytes were identified using the Genomatix Common Transcription Factor Binding Site software. TarBase was employed to identify the genes, among those commonly upregulated *in vivo* on Day 2 vs Day 0, and in ZZ monocytes treated *ex vivo* with AAT vs. untreated ZZ monocytes, that were reported to be targeted by the NFκB-regulated miRNAs. This list of genes was used as an input to perform overrepresentation analysis with WebGestalt (WEB-based Gene SeT Analysis Toolkit; http://www.webgestalt.org/option.php) using the whole human genome as the reference gene list.

The downregulated genes identified common to both Day 2 vs. Day 0 and ZZ + AAT vs. Control with an absolute fold change greater than 10 (n = 8) were uploaded to Enrichr (gene set enrichment analysis web server)^21^ and the gene ontology biological processes, molecular function and pathway involvement of the genes was explored. The significantly associated gene ontology terms (adjusted p-value < 0.05) were imported to REVIGO^22^ where they were clustered based on their relatedness and any redundancy was removed. Significantly enriched BioCarta, KEGG, NCI-Nature, Reactome and WikiPathways pathways, with adjusted p-value < 0.05 were exported from Enrichr and plotted.

A Gene Set Enrichment Analysis^35^ on downregulated genes after AAT augmentation therapy or following *in vitro* treatment with AAT using a NFκB gene signature was performed to support NFκB involvement.

### Statistical analysis

All analyses were performed using GraphPad PRISM 4.0 (San Diego, CA). Results are expressed as the mean ± SEM and were compared by Student *t* test or ANOVA with Bonferroni correction for multiple testing, as appropriate. Differences were considered significant at *p* ≤ 0.05 for individual comparisons.

### Data availability

Data generated or analysed during this study are included in this published article and its Supplementary Information files.

## Electronic supplementary material

Supplementary Information File

Dataset 1

Dataset 2

Dataset 3

Dataset 4

Dataset 5

Dataset 6
