# Supplementary material for: Alpha-1 antitrypsin augmentation therapy decreases miR-199a-5p, miR-598 and miR-320a expression in monocytes via inhibition of NFκB
Source: Sci Rep. 2017 Oct 23;7:13803. doi: 10.1038/s41598-017-14310-2 (PMC5653852; doi:10.1038/s41598-017-14310-2)
Supplement: Supplementary file 1 — Supplementary Information File [file 41598_2017_14310_MOESM1_ESM.pdf]

SUPPLEMENTARY Information File

Alpha-1 antitrypsin augmentation therapy decreases miR-199a-5p, miR-598 and miR-320a expression in monocytes via inhibition of NFκB

Tidi Hassan, Chiara de Santi, Catherine Mooney, Noel G. McElvaney, Catherine M. Greene

Supplementary Tables

Supplementary Table 1. Fold-change increase and decrease of miRNA expression in asymptomatic ZZ monocytes compared to MM monocytes.

miRNA	Fold-change increase	P-value	miRNA	Fold-change decrease	P-value
hsa-miR-769-5p	*21.8863582*	0.0003	*hsa-miR-328*	*-3.6587959*	0.0024
hsa-miR-572	14.9246721	0.0001	hsa-miR-363-3p	-6.3065355	0.0012
hsa-miR-92a	8.90749088	0.0009	*hsa-miR-1297*	*-4.1205028*	0.0003
hsa-miR-224	*14.0375707*	0.0017			
hsa-miR-548d-3p	*25.5685908*	0.0001			
hsa-miR-146a	*5.48083397*	0.0035			
hsa-miR-720	19.8319327	0.0001			
hsa-miR-34c-5p	*13.3300881*	0.0009			
hsa-miR-96	4.38590868	0.0012			
hsa-miR-598	16.9323611	0.0002			
hsa-miR-574-5p	27.5211135	0.0003			
hsa-miR-199a-5p	*41.8816313*	0.0001			
hsa-miR-101	26.021338	0.0008			
hsa-miR-626	7.73404922	0.0013			
hsa-miR-320a	12.427478	0.0001			
hsa-miR-383	4.96702074	0.0012			
hsa-miR-144-3p	*19.3295661*	0.0009			
hsa-miR-548f	*13.6282904*	0.0035			

Italicised: miRNAs that were also altered in symptomatic ZZs receiving AAT augmentation at Day 0 compared to asymptomatic ZZ monocytes.

Supplementary Table 2. Fold-change increase and decrease of miRNA expression in symptomatic ZZ monocytes receiving AAT augmentation therapy at Day 2 compared to asymptomatic ZZ monocytes not receiving AAT augmentation therapy.

miRNA	Fold-change increase	P-value	miRNA	Fold-change decrease	P-value
hsa-miR-148b	*6.63482193*	0.0001	*hsa-miR-769-5p*	*-8.1989062*	0.0002
hsa-miR-26b	3.71542585	0.0003	*hsa-miR-224*	*-17.181076*	0.0012
hsa-miR-30a-5p	4.6448871	0.0012	*hsa-miR-548d-3p*	*-27.651213*	0.0001
hsa-miR-655	*5.34608176*	0.0009	*hsa-miR-146a*	*-3.0137946*	0.0023
			hsa-miR-34c-5p	*-3.8785034*	0.0009
			hsa-miR-199a-5p	*-50.616489*	0.0001
			hsa-let-7d	*-3.3914874*	0.0024
			hsa-miR-144-3p	*-27.590396*	0.0001
			hsa-miR-548f	*-17.719249*	0.0012
			hsa-miR-92a	-10.942539	0.0003
			hsa-miR-598	-4.6110298	0.0009

Italicised: miRNAs that were also altered in symptomatic ZZs receiving AAT augmentation therapy at Day 0 compared to asymptomatic ZZ monocytes not receiving AAT augmentation therapy

Supplementary Table 3. Fold-change increase and decrease of miRNA expression in symptomatic ZZ monocytes receiving AAT augmentation therapy at Day 2 compared to symptomatic ZZ monocytes receiving AAT augmentation therapy at Day 0.

miRNA	Fold-change increase	P-value	miRNA	Fold-change decrease	P-value
hsa-miR-376c	3.129352	0.0012	hsa-miR-92a	-5.2475672	0.0005
hsa-miR-26b	3.71542585	0.0009	hsa-miR-598	-8.245107	0.0009
hsa-miR-30a-5p	4.6448871	0.0024	hsa-miR-199a-5p	-5.82474	0.0012
hsa-miR-655	5.34608176	0.0007	hsa-miR-133b	-3.2844425	0.0012
			hsa-miR-300	-3.5469325	0.0014
			hsa-miR-600	-5.9722382	0.0032
			hsa-miR-1297	-4.6856117	0.0021

Supplementary Table 4. List of the mRNAs that are (i) up-regulated in Day2 vs Day0, (ii) up-regulated in ZZ_AAT vs ZZ_CTRL and (iii) regulated by miR-598 according to TarBase.

**Gene ID (alias)**	**Description**	**FC in Day2 vs Day0**	**FC in ZZ_AAT vs ZZ_CTRL**
DLG5	discs large MAGUK scaffold protein 5	2.2614213	6.9214034
HSP90AA1	heat shock protein 90 alpha family class A member 1	26.019595	609.5365828
GGA2	golgi associated, gamma adaptin ear containing, ARF binding protein 2	17.2004941	5.795542

*FC=Fold Change

Supplementary Table 5. List of the mRNAs that are (i) up-regulated in Day2 vs Day0, (ii) up-regulated in ZZ_AAT vs ZZ_CTRL and (iii) regulated by miR-199a-5p according to TarBase.

**Gene ID (alias)**	**Description**	**[&]FC in Day2 vs Day0**	**[&]FC in ZZ_AAT vs ZZ_CTRL**
FN1	fibronectin 1	9.0196722	16.0721319
AKAP12	A-kinase anchoring protein 12	29.4065677	2.5605743
EIF4H	eukaryotic translation initiation factor 4H	2.6520298	26.6459802
ARCN1	archain 1	2.5290134	5.0986489
HNRNPU	heterogeneous nuclear ribonucleoprotein U	2.6073307	11.6291327
DCTPP1	dCTP pyrophosphatase 1	216.025783	3.0039321
EDEM1	ER degradation enhancing alpha-mannosidase like protein 1	10.2268348	2.5522264

*FC=Fold Change

[&]If more than one transcript variant from the same gene is up-regulated in the microarray set, the FC for the most common one is reported

Supplementary Table 6. List of the mRNAs that are (i) up-regulated in Day2 vs Day0, (ii) up-regulated in ZZ_AAT vs ZZ_CTRL and (iii) regulated by miR-320a according to TarBase.

**Gene ID (alias)**	**Description**	**[&]FC in Day2 vs Day0**	**[&]FC in ZZ_AAT vs ZZ_CTRL**
SCRN1	secernin 1	34.5998841	6.7471349
KLHL28	kelch like family member 28	2.1607617	602.3735028
MAN1A2	mannosidase alpha class 1A member 2	66.9875926	10.2397032
TRIM24	tripartite motif containing 24	16.9268545	4.2586702
RASA2	RAS p21 protein activator 2	20.4163449	2.0645528
STAT5B	signal transducer and activator of transcription 5B	99.9598132	2.8179441
SAMD4B	sterile alpha motif domain containing 4B	22.087991	22.2340445
HNRNPU	heterogeneous nuclear ribonucleoprotein U	2.6073307	11.6291327
LBR	lamin B receptor	5.7069028	5.8174141
SMAD3	SMAD family member 3	10.2183333	301.9091351
DCAF12	DDB1 and CUL4 associated factor 12	16.9274916	15.4527146
ANGEL1	angel homolog 1	3.1654158	13.9052521
HOXA7	homeobox A7	9.6598354	2.9732946
ATPAF2	ATP synthase mitochondrial F1 complex assembly factor 2	8.3170333	129.2621157
ABHD16A	abhydrolase domain containing 16A	5.6256781	2.8979787
RAB3IP	RAB3A interacting protein	2.2447634	9.2491257
YWHAQ	tyrosine 3-monooxygenase/tryptophan 5-monooxygenase activation protein theta	23.4439701	3.1623784
RBM38	RNA binding motif protein 38	2.7218225	3.1350664
POLR2F	RNA polymerase II subunit F	6.621183	23.8952226
EIF4H	eukaryotic translation initiation factor 4H	2.6520298	26.6459802
PCBP2	poly(rC) binding protein 2	5.9098028	3.5195452
KIAA1598 (*SHTN1*)	shootin 1	4.6074493	2.5273061
NXT2	nuclear transport factor 2 like export factor 2	115.7640508	74.4001178
AUTS2	AUTS2, activator of transcription and developmental regulator	132.5370956	2.5713357
PDLIM5	PDZ and LIM domain 5	7.8777848	3.1507046
KDM5C	lysine demethylase 5C	17.115114	2.5289403
FAM208B	family with sequence similarity 208 member B	2.5127794	3.8634122
KANSL1	KAT8 regulatory NSL complex subunit 1	55.765038	9.6229618
TJP1	tight junction protein 1	17.0916396	20.050026
NBEAL2	neurobeachin like 2	25.7004462	3.4311614

COTL1	coactosin like F-actin binding protein 1	49.8351235	8.7462755
NFIB	nuclear factor I B	13.6029533	3.1346144
GLUL	glutamate-ammonia ligase	5.2847298	2.0580822
UBAP2L	ubiquitin associated protein 2 like	57.2601681	573.7182497
TSC22D4	TSC22 domain family member 4	589.1975822	489.5328959
TMEM194A (*NEMP1*)	nuclear envelope integral membrane protein 1	2.3244861	2.921866
DYRK1A	dual specificity tyrosine phosphorylation regulated kinase 1A	3.7361387	2.1959366
NAA25	N(alpha)-acetyltransferase 25, NatB auxiliary subunit	160.2449853	11.9818909
RPS27	ribosomal protein S27	2.0046161	6.9838346

*FC=Fold Change

[&]If more than one transcript variant from the same gene is up-regulated in the microarray set, the FC for the most common one is reported

Supplementary Table 7. Pathway analysis of upregulated mRNAs in Supplementary Tables 4-6.

KEGG pathway	Genes mapped in the pathway	P-value	£Adjusted P-value
AGE-RAGE signalling pathway in diabetic complications	*FN1*, *SMAD3*, *STAT5B*	$1.33x10^{-3}$	0.238
Th17 cell differentiation	*HSP90AA1*, *SMAD3*, *STAT5B*	$1.57x10^{-3}$	0.238
Hepatitis B	*YWHAQ*, *SMAD3*, *STAT5B*	$3.81x10^{-3}$	0.384
Protein processing in endoplasmic reticulum	*MAN1A2*, *HSP90AA1*, *EDEM1*	$5.46x10^{-3}$	0.413
Viral carcinogenesis	*YWHAQ*, *RASA2*, *STAT5B*	$9.78x10^{-3}$	0.434

£Benjamini-Hochberg corrected P-value for multiple testing (FDR)

Supplementary Figure 1. “Interactive graph” view from REVIGO showing significantly altered GO terms (A), Biological Processes and (B), Molecular Function. The bubble colour indicates the p-value generated by the Enrichr analysis (darker implies smaller p-value) and the bubble size indicates the frequency of the GO term. Highly similar GO terms are linked by edges in the graph, where the line width indicates the degree of similarity.

A.

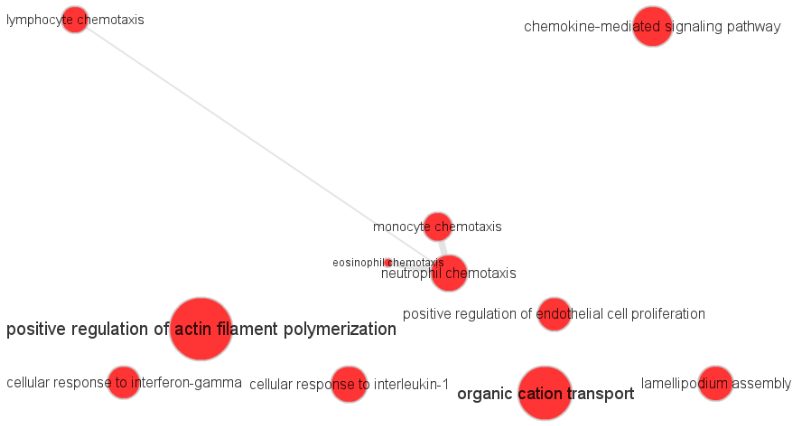

B.

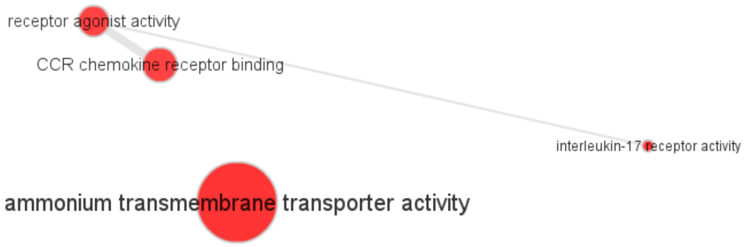

Supplementary Figure 2. Bar plots showing the −log10(p-value) of significantly enriched (adjusted p-value < 0.05) (A) BioCarta, (B) KEGG, (C) NCI-Nature, (D) Reactome and (E) WikiPathways pathways. The number of targets in a given pathway is shown in parentheses.

A. BioCarta

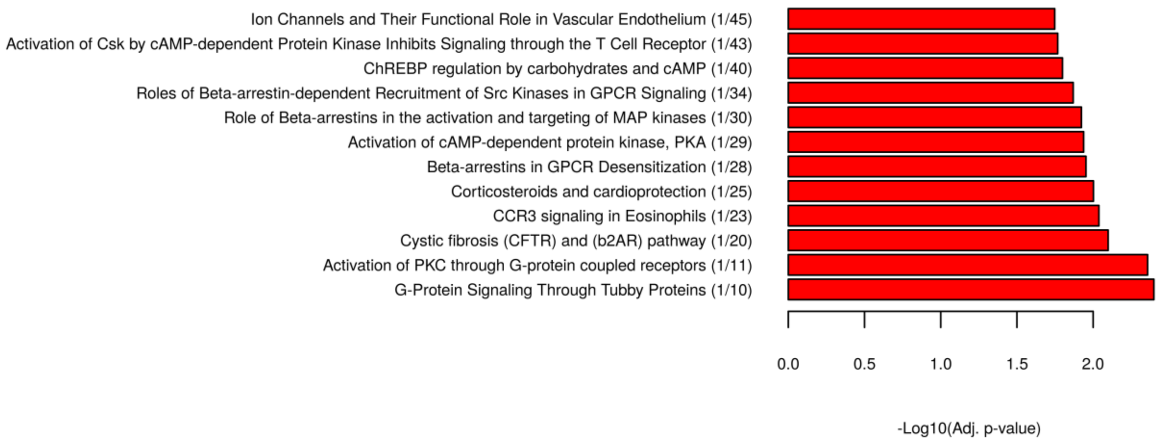

B. KEGG

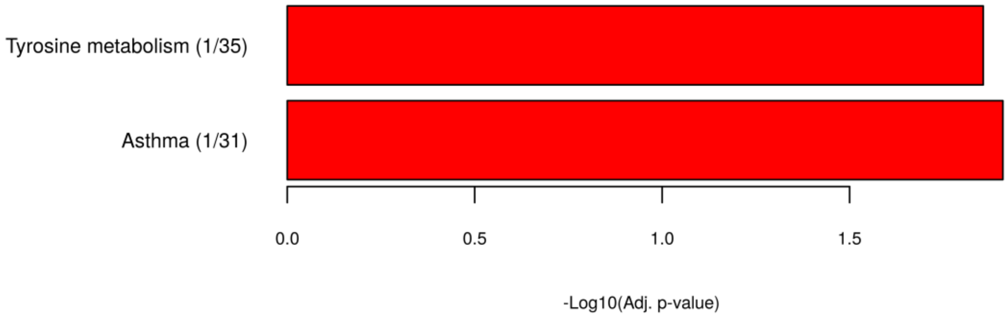

C. NCI-Nature

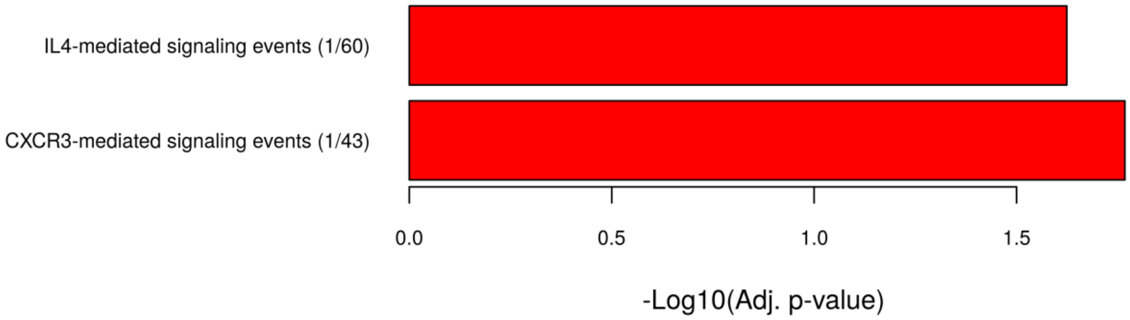

D. Reactome

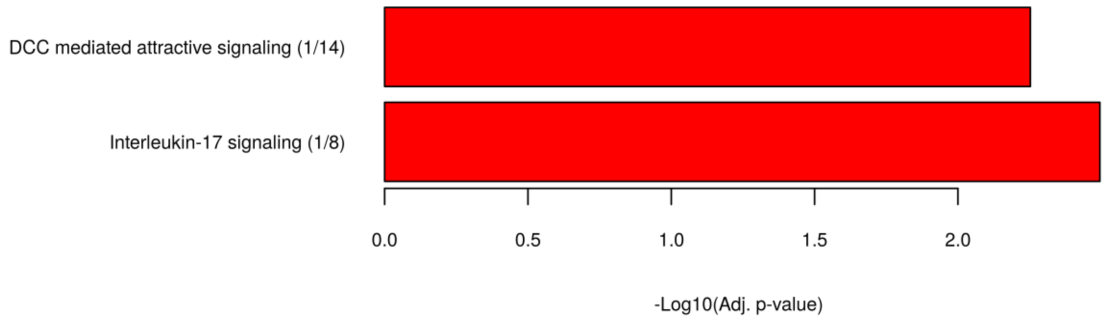

E. WikiPathways

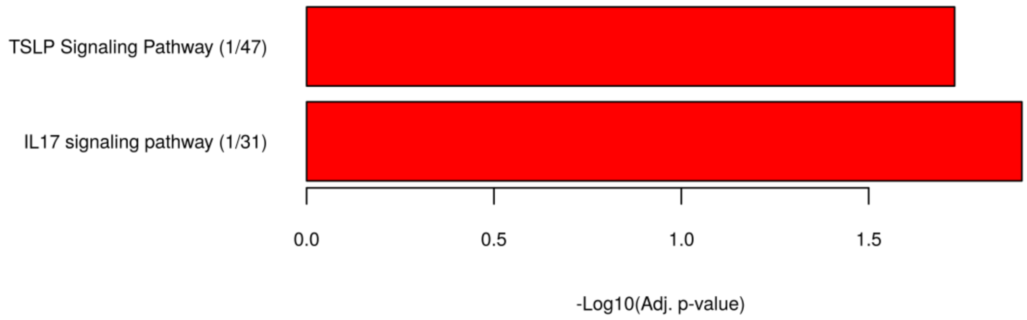

Supplementary Figure 3.
Summary heat maps of (*A)*, miR-598, miR-199a-5p, miR-320a and miR-30a, or *(B),* 60 miRNAs profiled using the nCounter miRNA Expression Assay in THP-1 monocytic cell lines (1x10^5 cells in triplicate), asymptomatic MM and ZZ monocytes and in ZZ monocytes of individuals receiving AAT augmentation therapy (Day 0; pre-infusion, Day 2; 48 hours post-infusion) (n=3 in each group). Red, increase in expression (>1.5 fold); green, decrease in expression (>1.5 fold) relative to background corrections for each miRNA as instructed in the nCounter Data Analysis Guidelines. 731 miRNAs were profiled using the nCounter miRNA Expression Assay. Altered miRNAs were identified with an average difference of greater or less than 1.5 fold when asymptomatic MM was compared to ZZ monocytes, and when asymptomatic ZZ was compared to ZZ monocytes receiving AAT at Day 0. Red, implicates values above 10 whilst green implicates values below 10 in expression relative to background corrections for each miRNA as instructed in the nCounter Data Analysis Guidelines.

A.

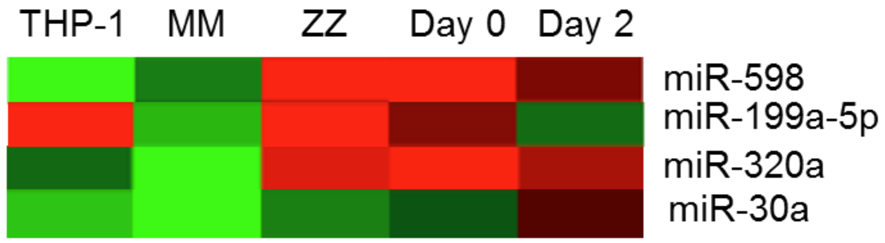

B.

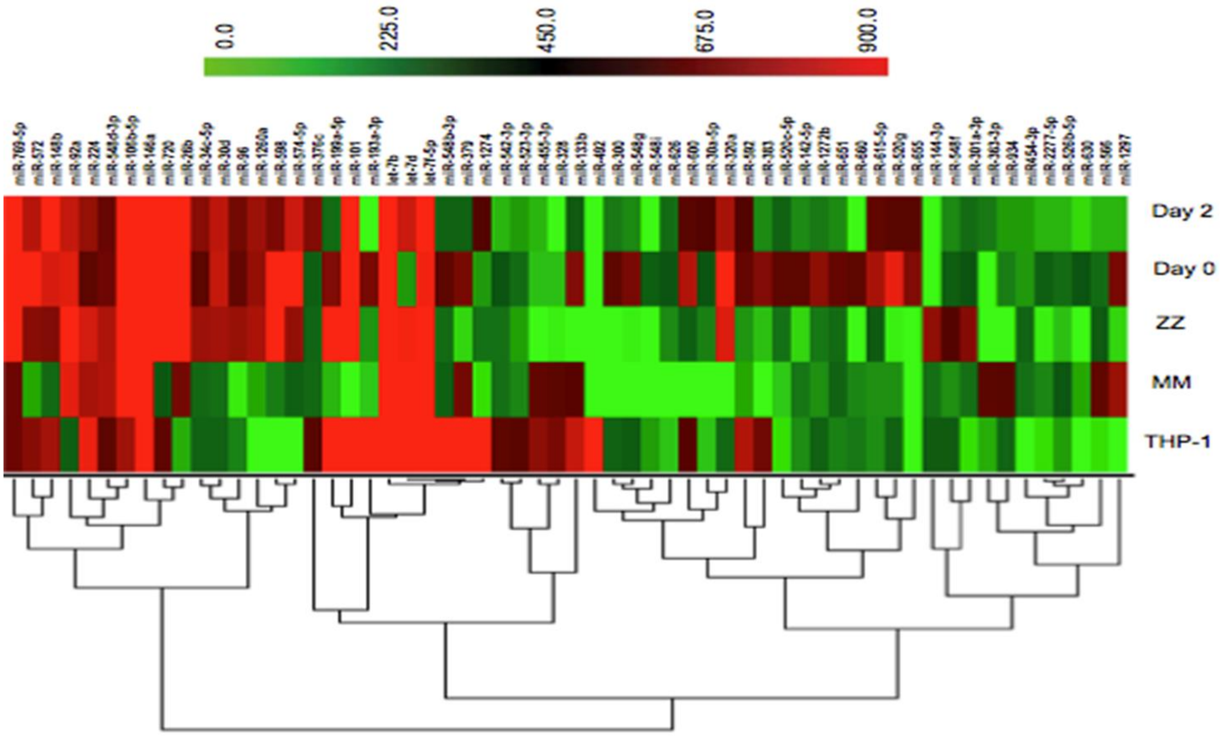

Supplementary Figure 4.
Uncropped western blots of Figure 5B. The images in Fig. 5B correspond to ‘preAATD’ and ‘AATD2’, respectively.

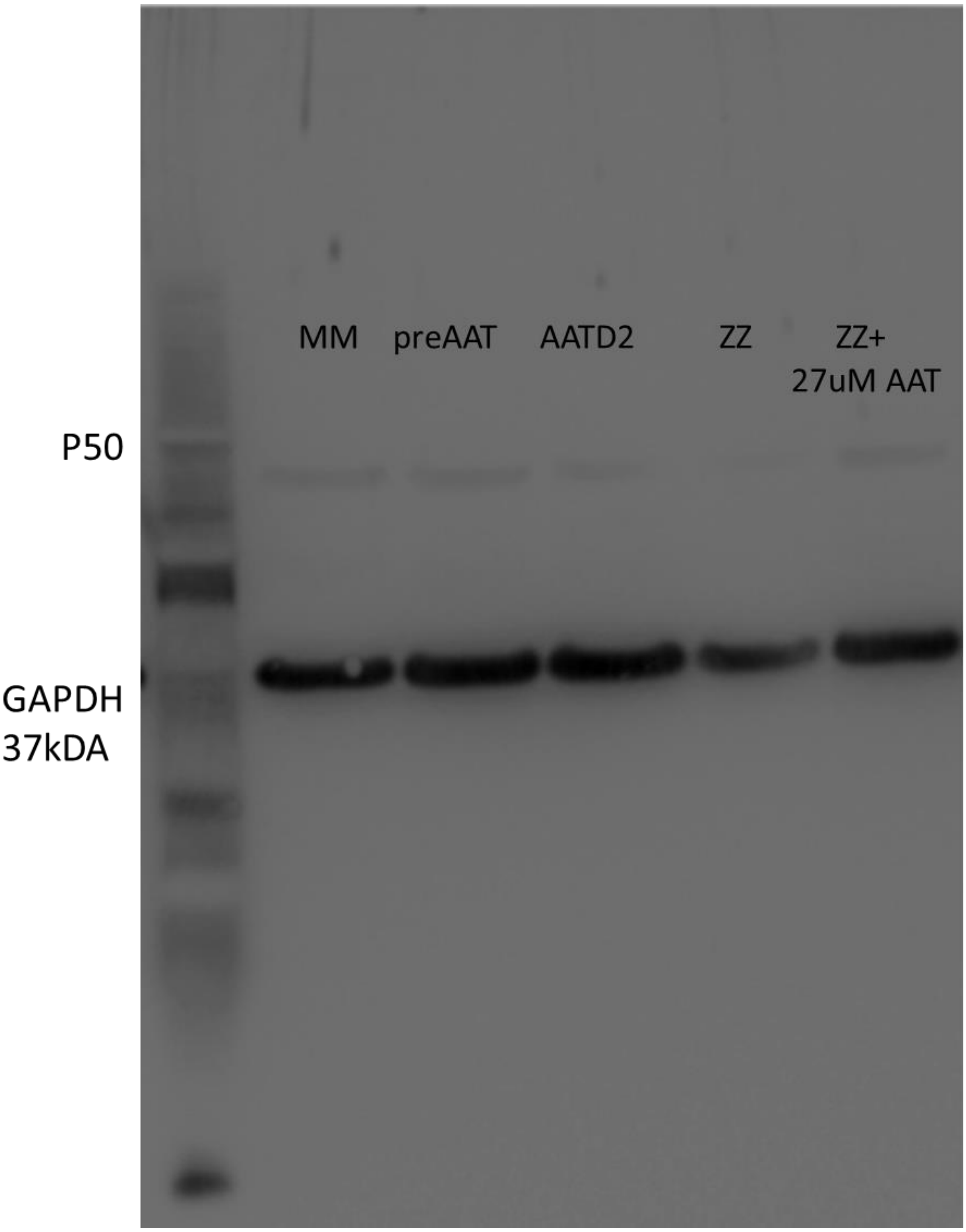

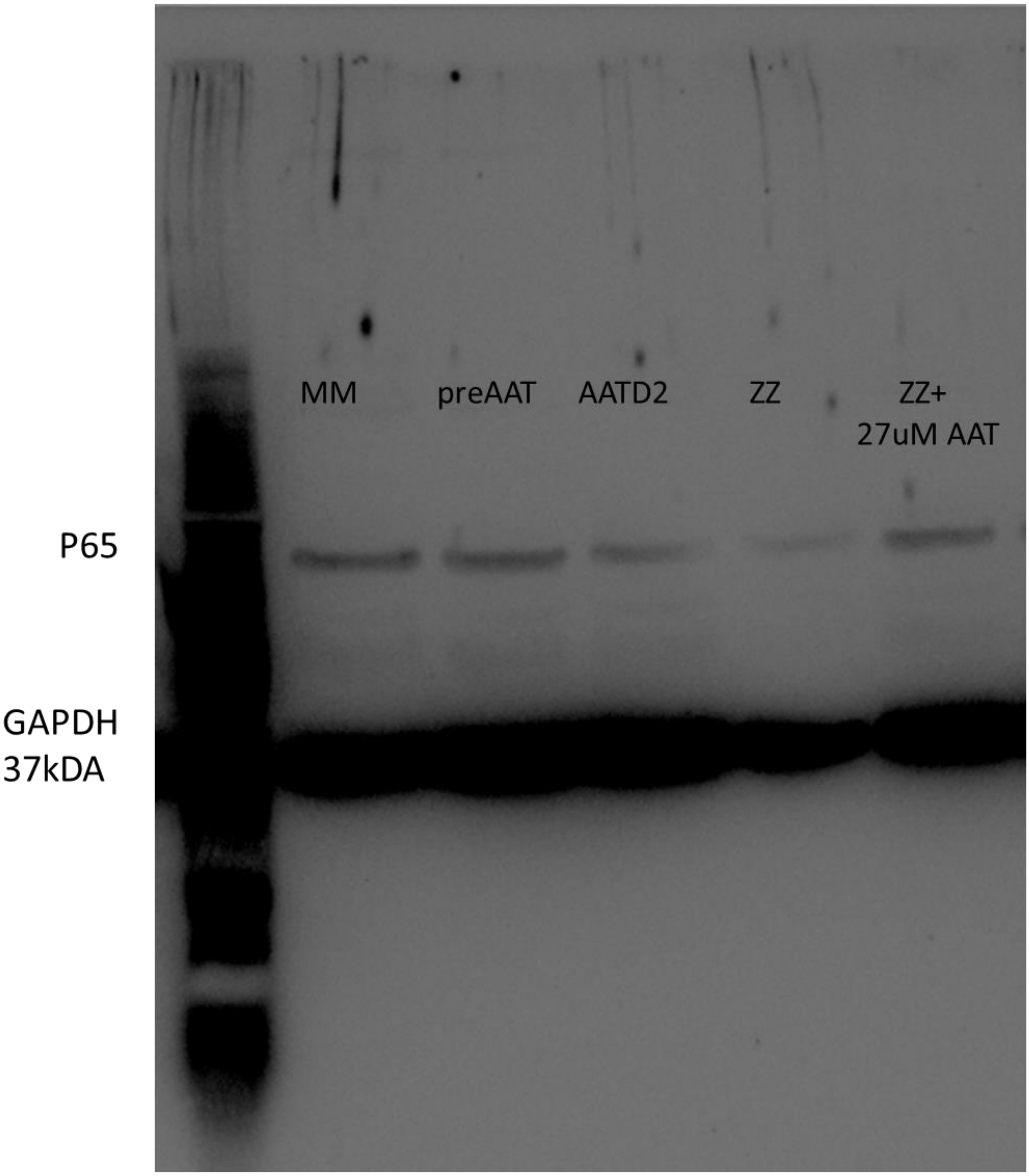
P65
GAPDH
37kDA
MM
preAAT
AATD2
ZZ
ZZ+
27uM AAT
